# Predicting Norovirus in the United States Using Google Trends: Infodemiology Study

**DOI:** 10.2196/24554

**Published:** 2021-09-29

**Authors:** Kai Yuan, Guangrui Huang, Lepeng Wang, Ting Wang, Wenbin Liu, Haixu Jiang, Albert C Yang

**Affiliations:** 1 School of Life Sciences, Beijing University of Chinese Medicine Beijing China; 2 School of Humanities Beijing University of Chinese Medicine Beijing China; 3 Digital Medicine Center National Yang Ming Chiao Tung University Taiwan Republic of China; 4 Department of Medical Research Taipei Veterans General Hospital Taiwan Republic of China; 5 Division of Interdisciplinary Medicine and Biotechnology Beth Israel Deaconess Medical Center/Harvard Medical School Boston, MA United States

**Keywords:** norovirus, Google Trends, correlation, outbreak, predictors

## Abstract

**Background:**

Norovirus is a contagious disease. The transmission of norovirus spreads quickly and easily in various ways. Because effective methods to prevent or treat norovirus have not been discovered, it is important to rapidly recognize and report norovirus outbreaks in the early phase. Internet search has been a useful method for people to access information immediately. With the precise record of internet search trends, internet search has been a useful tool to manifest infectious disease outbreaks.

**Objective:**

In this study, we tried to discover the correlation between internet search terms and norovirus infection.

**Methods:**

The internet search trend data of norovirus were obtained from Google Trends. We used cross-correlation analysis to discover the temporal correlation between norovirus and other terms. We also used multiple linear regression with the stepwise method to recognize the most important predictors of internet search trends and norovirus. In addition, we evaluated the temporal correlation between actual norovirus cases and internet search terms in New York, California, and the United States as a whole.

**Results:**

Some Google search terms such as gastroenteritis, watery diarrhea, and stomach bug coincided with norovirus Google Trends. Some Google search terms such as contagious, travel, and party presented earlier than norovirus Google Trends. Some Google search terms such as dehydration, bar, and coronavirus presented several months later than norovirus Google Trends. We found that fever, gastroenteritis, poison, cruise, wedding, and watery diarrhea were important factors correlated with norovirus Google Trends. In actual norovirus cases from New York, California, and the United States as a whole, some Google search terms presented with, earlier, or later than actual norovirus cases.

**Conclusions:**

Our study provides novel strategy-based internet search evidence regarding the epidemiology of norovirus.

## Introduction

Norovirus, also named stomach bug or winter vomiting bug, is a kind of contagious virus leading to gastroenteritis [1]. It is responsible for almost 20% of acute gastroenteritis cases worldwide [2]. Especially in cruise ships, norovirus is the most common cause of gastrointestinal illness [3]. The symptoms of norovirus include diarrhea, nausea, and vomiting, and it can be transmitted by contaminated water, contaminated food, and person-to-person contact [4,5]. Polymerase chain reaction and enzyme-linked immunosorbent assay are useful methods to diagnose norovirus infection [6,7]. Thus far, there have been no specific drugs to treat norovirus infection. In the prevention of norovirus, hand washing and disinfectants are important and effective methods. In addition, avoiding contaminated water and contaminated food in restaurants are useful methods to prevent norovirus.

It is important to rapidly recognize and report norovirus infection. In the United States, the US Centers for Disease Control and Prevention (CDC) report and monitor the outbreak of gastroenteritis with an internet search system. In addition, other surveillance systems, such as the Foodborne Diseases Active Surveillance Network, can be used to report the trends of foodborne disease outbreaks [8]. However, these surveillance and reporting systems have a lag of 1 to 2 weeks after an outbreak due to the reporting and verification procedure. Internet search has been a useful method for people to access information immediately. Infodemiology is an emerging field based on public attention, knowledge, opinion, etc [9]. Data from infodemiology could be acquired and analyzed to enhance public health [10] and predict outbreaks of disease such as influenza [11]. In addition, infodemiology could also be used to monitor foodborne disease, gastroenteritis, dengue, etc [12]. Apart from Google search, search engines from other platforms including Facebook and Twitter can benefit infoveillance [13]. Yang et al [14-16] revealed the correlation between internet search trends and suicide incidence and depression. In addition, internet searches have been used to monitor infectious diseases, including influenza and Lyme disease [17,18]. Mavragani et al [19] analyzed 104 published papers published from 2006 to 2016 related to Google Trends and found that the monitoring of online queries can provide insight into human behavior. In addition, Nuti et al [20] analyzed 70 papers published from 2009 to 2013 and discovered the topic domains related to Google Trends were general population behavior, infectious disease, noncommunicable diseases, mental health, and substance use.

In this study, we discovered a correlation between internet search terms and norovirus. The search trend of Google Insight was extracted and used in our study. Certain keywords in the Google search trend included “diarrhea,” “vomiting,” “fever,” etc. These keywords could reflect the characteristics of norovirus infection. We also conducted a monthly time series analysis to discover the relationship between internet search terms and norovirus. We hoped to provide new insight to reveal the epidemic and outbreak of norovirus using internet search data.

## Methods

### Internet Search Approach

The internet search data relating to norovirus were obtained from Google Trends [21]. Google Trends is a useful website to discover search queries from various regions and countries. This tracking system could also provide detailed analysis of Google searches. Google Trends could provide long time series and geographical search locations. Google Trends is in widespread use to investigate medical topics. We downloaded the data of various terms in monthly granularity. The normalization of data indicated that the values vary from 0 to 100. The value 0 indicated very low search volumes that are not included in the results, while the value 100 indicated very high search volumes. The data are retrieved directly from the Google Trends Explore page in .csv format after keywords are entered and the region, period, and category are selected (see Multimedia Appendix 1) [22]. We chose geographic locations for search volumes as the states of New York and California and the United States as a whole. A time range from January 1, 2004, to December 31, 2018, was chosen. The category was limited to Health to avoid unrelated results of Google searches. We used 50 search terms to reflect a broad sense of norovirus: “norovirus,” “gastroenteritis,” “diarrhea,” “vomiting,” “dehydration,” “contaminated,” “norovirus infection,” “watery diarrhea,” “contagious,” “contaminated water,” “Norwalk virus,” “noroviruses,” “acute gastroenteritis,” “stomach flu,” “viral gastroenteritis,” “winter vomiting disease,” “stomach bug,” “travel,” “party,” “barbecue,” “cruise,” “oyster,” “bar,” “restaurant,” “wedding,” “hotel,” “motel,” “virus,” “infectious,” “outbreak,” “rotavirus,” “coronavirus,” “influenza,” “food poisoning,” “incubation period,” “fever,” “poison,” “CDC,” “vaccine,” “Chipotle,” “ship,” “hand sanitizer,” “wash hand,” “flu symptom,” “streptococcus,” “antibiotics,” “candidiasis,” “otitis media,” “skin rash,” and “coxsackie virus.” These terms were chosen to indicate the related symptoms of and risk factors for norovirus. These terms could provide positive and negative information about norovirus. The Google Trends data were exhibited on a monthly basis. No patient records or personal information were included in the search results of Google Trends. Google Trends normalizes search data to make comparisons between terms easier. Search results are normalized to the time and location of a query. Each data point is divided by the total searches of the geography and time range it represents to compare relative popularity. The resulting numbers are then scaled on a range of 0 to 100 based on a topic’s proportion to all searches on all topics [23].

### Actual Norovirus Cases Acquisition

Actual norovirus cases from California, New York, and the country as a whole were obtained from the National Outbreak Reporting System (NORS) at the CDC [24,25]. We limited the etiology to “norovirus” ranging from 2004 to 2018. In addition, the illnesses in every month varied from January 2004 to December 2018. Cross-correlation analysis was conducted to reveal the relationship between actual norovirus cases and internet search terms.

### Statistical Analysis

SPSS for Windows (version 20.0, IBM Corp) was used for statistical analysis. A 2-stage modeling process was conducted to evaluate the correlation between norovirus and internet search trends. Cross-correlation analysis was conducted to reveal the relationship between 2 parameters. Cross-correlation is a method to discover the correlation between 2 time series parameters contemporaneously and with various lagged values. Cross-correlation could help researchers understand the relationship between 2 parameters. In addition, cross-correlation analysis could also help researchers understand whether one parameter was preceded by or followed another. Thus, cross-correlation could estimate the time lag of norovirus and other internet search trend terms. The results of cross-correlation were reported in our study.

We used a multiple linear regression method to recognize the most important predictors of internet search trends. Multiple linear regression was used in the second stage to analyze the internet search terms. A stepwise method in multiple linear regression was conducted to discover significant parameters correlated with norovirus. It also could help researchers evaluate the variation in monthly norovirus counts explained with significant parameters. A stepwise method could reduce the collinearity that internet search might be intercorrelated. The variance inflation factor was used to evaluate the collinearity of internet search data. Those terms with variance inflation factor >10 were excluded from the model. The summary of multiple linear regression was reported as results. A 2-tailed *P*<.05 was regarded as significant in all analyses.

## Results

### Basic Features Description

**Figure 1:**
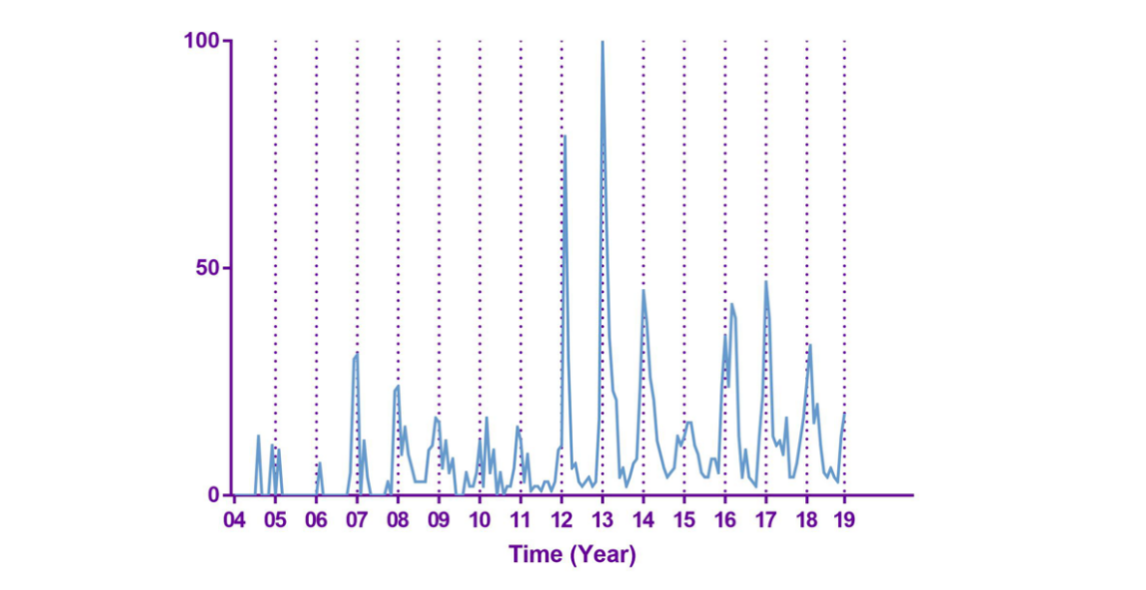
The basic features of the norovirus Google search.

**Figure 2:**
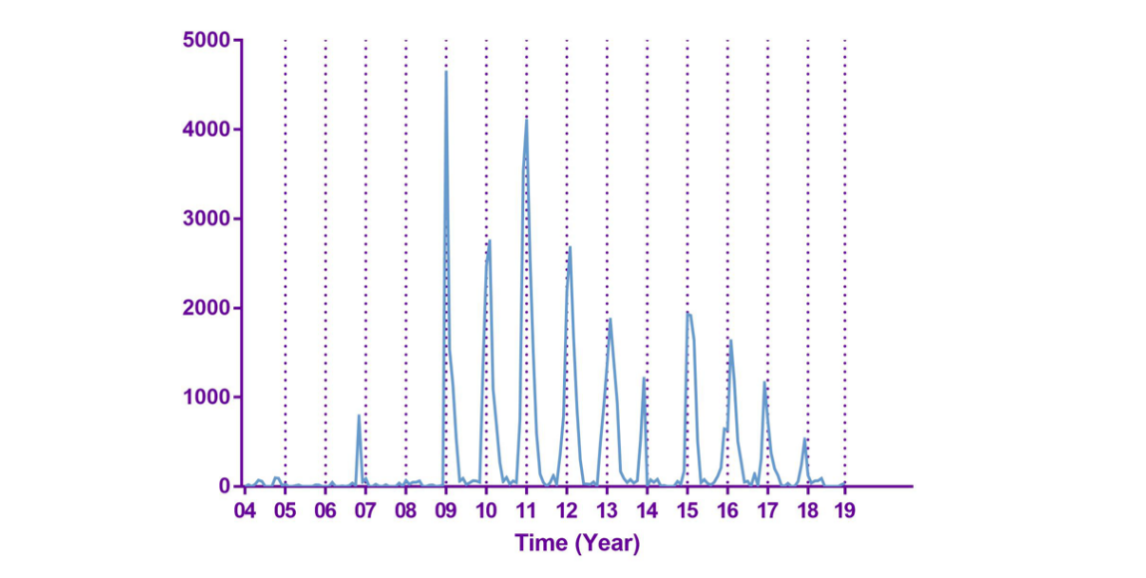
The basic features of actual norovirus cases.

The results of Google Trends in the state of New York from January 2004 to December 2018 were downloaded in our study. Except for the search term “norovirus,” a total of 50 keywords from Google Trends were downloaded. Visual inspection indicated that the value of norovirus in Google Trends peaked around December to February in most years (Figure 1). The basic features of actual norovirus cases are shown in Figure 2.

### Temporal Correlation in New York

We used cross-correlation analysis to discover the temporal correlation between norovirus and other terms. The internet search terms were included for cross-correlation analysis with norovirus. Multimedia Appendix 2 indicates the results of the cross-correlation of norovirus and other internet search terms. The gray markup shows the lead pattern for norovirus and other internet search terms. The search terms “gastroenteritis,” “watery diarrhea,” “stomach flu,” “winter vomiting disease,” “stomach bug,” and “food poisoning” were coincided with norovirus in Google Trends. The search terms of “contagious,” “travel,” “party,” “restaurant,” “wedding,” “hotel,” “infectious,” and “poison” presented earlier than norovirus Google Trends. The search terms of “dehydration,” “bar,” “Chipotle,” and “coronavirus” presented several months later than norovirus Google Trends.

After discovering the temporal correlation between norovirus and other terms, we used multiple linear regression with the stepwise method to recognize the most important predictors of internet search trends and norovirus. The search terms that did not have a relationship with norovirus were excluded from the cross-correlation analysis. The internet search terms in Multimedia Appendix 2 were included for multiple linear regression analysis with a norovirus search. The results indicated that searches for “stomach flu,” “stomach bug,” “winter vomiting disease,” “gastroenteritis,” and “cruise” were significant predictors of norovirus search and accounted for 55% (*R*^2^) of the variation in norovirus search (Table 1).

**Table 1:** Stepwise regression model of norovirus and correlated internet search trends in New York State.

Search term	Model summary		
	Standardized β	SE	*P* value
Stomach flu	0.24	0.06	<.001
Stomach bug	0.29	0.06	<.001
Winter vomiting disease	0.24	0.05	<.001
Gastroenteritis	0.13	0.05	.01
Cruise	0.16	0.07	.03

Norovirus also refers to stomach flu, stomach bug, or winter vomiting disease. We excluded these terms to identify the possible predictors of norovirus. Therefore, “stomach flu,” “stomach bug,” “winter vomiting disease,” and “norovirus infection” were excluded in this multiple linear regression analysis. The results showed that searches for “fever,” “gastroenteritis,” “poison,” “cruise,” “wedding,” “watery diarrhea,” and “acute gastroenteritis” accounted for 40% (*R*^2^) of the variation in the norovirus search (Table 2). We found that the symptoms of gastroenteritis, including vomiting and watery diarrhea, were important factors that were significantly correlated with norovirus. In addition, activities such as cruise and wedding were important in norovirus outbreak.

**Table 2:** Stepwise regression model of norovirus and correlated internet search trends excluded alternate names of norovirus in New York State.

Search term	Model summary		
	Standardized β	SE	*P* value
Fever	0.33	0.08	<.001
Gastroenteritis	0.19	0.06	.001
Poison	–0.16	0.05	.002
Cruise	0.32	0.10	.001
Wedding	–0.34	0.11	.002
Watery diarrhea	0.09	0.04	.02
Acute gastroenteritis	0.09	0.04	.04

### Identifying Predictors of Actual Norovirus Cases

In this study, we evaluated the temporal correlation between actual norovirus cases in New York and internet search terms in New York. The data from actual norovirus cases were downloaded from the CDC. As shown in Multimedia Appendix 3, the search terms “norovirus,” “gastroenteritis,” “norovirus infection,” “contagious,” and “acute gastroenteritis” were coincided with actual norovirus cases in New York. The search terms “travel,” “party,” “barbecue,” and “cruise” presented earlier than actual norovirus cases in New York. The search terms “dehydration,” “winter vomiting disease,” “outbreak,” and “rotavirus” presented one or several months later than actual norovirus cases in New York.

Furthermore, we evaluated the temporal correlation between actual norovirus cases in California with internet search terms in California. The search terms including “barbecue,” “oyster,” “bar,” and “infectious” were presented one or several months earlier than actual norovirus cases in California. The search terms including “diarrhea,” “vomiting,” “contagious,” “contaminated water,” and “Norwalk virus” were presented one or several months later than actual norovirus cases in California. The detailed information is shown in Multimedia Appendix 4.

In addition to New York and California, we also evaluated the temporal correlation between actual norovirus cases with internet search terms within the United States. The search terms “vomiting,” “contaminated,” “norovirus infection,” and “contagious” coincided with actual norovirus cases in the United States. Search terms including “travel,” “party,” “cruise,” “restaurant,” and “wedding” presented one or several months earlier than actual norovirus cases in the United States. The search terms “gastroenteritis,” “diarrhea,” “dehydration,” and “watery diarrhea” presented one or several months later than actual norovirus cases in the United States. The detailed information is shown in Multimedia Appendix 5.

## Discussion

### Principal Findings

In our study, we found that norovirus was correlated with the search terms “gastroenteritis,” “diarrhea,” “vomiting,” “dehydration,” “contagious,” “stomach flu,” “stomach bug,” “restaurant,” and “food poisoning.” In the total related search terms, gastroenteritis, diarrhea, watery diarrhea, fever, vomiting, and dehydration were typical symptoms of norovirus. Stomach flu, winter vomiting disease, and stomach bug are other names of norovirus. Restaurant and bar were food service settings that occurred with norovirus outbreaks. Chipotle Mexican Grill is a specific restaurant that has experienced norovirus outbreaks in the United States. Apart from the previous search terms, streptococcus and coxsackievirus were related to norovirus. Infections with streptococcus and coxsackievirus have characteristics similar to those of norovirus, such as fever and contagious features.

Infodemiology could be discovered and analyzed in near real time. Many disease outbreaks could be predicted and monitored by infodemiology. The applications of infodemiology included queries analysis with internet search engines, people’s status updates monitor, publications related to public health monitors, etc. Patthi et al [26] used Google Trends from 2004 to 2016 to investigate the global search trends of oral problems. Their method provided an insight to analyze and compare oral disease over time on a mass population. Shimkhada et al [27] used Twitter Chat to identify barriers and responsive policy of patients with metastatic breast cancer care. Klein et al [28] conducted Twitter data to track the spread of COVID-19. Johnson et al [29] performed Google Trends to monitor sexually transmitted infections Chicago. Thus, infodemiology could provide valuable insights into health-related behavior of populations [9].

In this study, we discovered the correlation between norovirus and other search terms in Google search trends. Norovirus is a kind of infectious disease leading to diarrhea and vomiting [30]. Extremely or chronic diarrhea and vomiting can lead to dehydration in some cases of norovirus illness patients [31,32]. There are various transmission methods for norovirus [33]. First, noroviruses are transmitted to healthy people by contaminated food [34,35]. Foods such as oysters or fruits might be contaminated by water containing norovirus particles [36]. Second, noroviruses can be transmitted to healthy people by contaminated water [37]. Last, the norovirus could spread to healthy people by surfaces contaminated by infected people [38]. Therefore, norovirus particles easily infect healthy people via contaminated food, water, and surfaces. Because an effective vaccine to prevent norovirus has not been discovered, it is important for healthy people to prevent norovirus infection by washing hands thoroughly [39]. The transmission of norovirus could be effectively decreased by practicing proper hand hygiene, preparing food safely, sterilizing surfaces, and washing laundry thoroughly. In this study, we used reasonable prior knowledge of norovirus-related risks to define our list of search terms. In order to cover a wider range of norovirus-related search, we adopted a loose criteria to choose the terms used in this paper that can be used to predict norovirus outbreak.

This study discovered the temporal correlation between norovirus and other terms. Dehydration would be caused by chronic diarrhea or vomiting in older adults or young children. Thus, the search terms including “dehydration” were presented several months later than norovirus in Google Trends. Interestingly, the search term “contagious” was presented earlier than “norovirus” in Google Trends. This indicated that people might contact some contaminated water or food. In addition, people might contact directly with other people who are infected with norovirus. Therefore, they search the term “contagious” before “norovirus.” Apart from typical symptoms, the stepwise regression model showed that internet searches for “cruise” and “wedding” accounted for predictors in the norovirus search. Thus, the internet search terms of some activities might be useful indicators of norovirus outbreaks. In these cases, internet search might occur several months earlier than actual activity for the purpose of reservation and planning ahead of these activities.

Apart from norovirus Google Trends, we also used the data of actual norovirus cases to validate the conclusion. In New York, the cross-correlation analysis of actual norovirus cases and internet search terms showed that the results were similar to norovirus in Google Trends. The Google Trends results of “travel,” “party,” and “wedding” presented one or several months earlier than actual norovirus cases. This result indicated that the search terms “party” and “travel” might be factors of norovirus outbreaks. “Party” and “travel” would lead to people gathering and cause norovirus transmission. This search strategy could be a useful method to predict and monitor outbreaks of norovirus in New York and the United States. Later, we also evaluated the temporal correlation between actual norovirus cases in California with internet search terms in California. However, the results of California were different from those of New York and the United States. The geographic area of California was much bigger than New York. The California illnesses in every norovirus outbreak might be less than New York. It could explain the reason for the difference between New York and California.

### Limitations

There are some limitations in this work. First, we tried to discover the correlation between internet search terms and norovirus infection in New York, California, and the United States. Other countries in the world were not included in this study. The results of the United States with Google Trends might not be applied to other countries worldwide. Second, the internet search engine Google was used in this study, other internet search engines and social networking services such as Bing, Twitter, and Facebook were not included in this study. In the future, the social networking services could be included to evaluate outbreaks of norovirus. Last, the norovirus data and Google Trends data were available monthly. Thus, we have not calculated the correlation between daily data on norovirus infections or hospitalizations with Google Trends data in this study. In the future, the correlation between daily data on norovirus infections or hospitalizations with Google Trends data could be explored if the daily data are available.

### Conclusions

In this study, we used Google search trends to investigate the correlation between internet searches and norovirus. The data were downloaded from Google Trends and NORS at the CDC. We used cross-correlation analysis to discover the temporal correlation between norovirus and other terms. We found that search trend data from Google is useful to predict norovirus outbreak. Our study provides a novel strategy based on internet search data to investigate the infodemiology of norovirus and monitor the outbreak of norovirus in the future.

## Appendix

Multimedia Appendix 1 The average CSV about the search terms in Google Trends.

Multimedia Appendix 2 Cross-correlation analysis of norovirus and Internet search terms–New York.

Multimedia Appendix 3 Cross-correlation analysis of actual norovirus cases and Internet search terms–New York.

Multimedia Appendix 4 Cross-correlation analysis of actual norovirus cases and Internet search terms–California.

Multimedia Appendix 5 Cross-correlation analysis of actual norovirus cases and Internet search terms–US.

## Abbreviations

CDC: Centers for Disease Control and Prevention
NORS: National Outbreak Reporting System
