# Supplementary material for: Predicting Norovirus in the United States Using Google Trends: Infodemiology Study
Source: J Med Internet Res. 2021 Sep 29;23(9):e24554. doi: 10.2196/24554 (PMC8515228; doi:10.2196/24554)
Supplement: Multimedia Appendix 1 [file jmir_v23i9e24554_app1.docx]

**Multimedia Appendix 1. The average csv about the search terms in Google Trends**

| Search terms | New York | California | USA |
| --- | --- | --- | --- |
| norovirus | 10.0 | 8.1 | 13.6 |
| gastroenteritis | 26.3 | 49.3 | 49.3 |
| diarrhea | 49.6 | 48.1 | 47.6 |
| vomiting | 52.7 | 51.0 | 56.3 |
| dehydration | 52.0 | 56.7 | 54.6 |
| contaminated | 13.2 | 31.8 | 39.2 |
| Norovirus infection | 2.5 | 7.2 | 17.4 |
| watery diarrhea | 26.0 | 26.3 | 37.1 |
| contagious | 41.3 | 42.5 | 42.4 |
| contaminated water | 4.6 | 8.0 | 21.7 |
| Norwalk virus | 7.7 | 6.8 | 13.1 |
| Noroviruses | 1.4 | 1.9 | 6.0 |
| acute gastroenteritis | 10.4 | 11 | 19.5 |
| stomach flu | 24.7 | 32.6 | 26.1 |
| viral gastroenteritis | 6.8 | 7.4 | 34.1 |
| stomach bug | 17.4 | 20.0 | 22.4 |
| travel | 57.6 | 53.9 | 53.8 |
| party | 63.7 | 60.0 | 64.8 |
| barbecue | 41.9 | 51.1 | 51.7 |
| cruise | 48.2 | 41.1 | 49.1 |
| oyster | 62.5 | 63.2 | 65.3 |
| bar | 74.2 | 76.4 | 74.1 |
| restaurant | 76.4 | 81.3 | 72.5 |
| wedding | 47.8 | 45.1 | 45.9 |
| hotel | 61.7 | 61.1 | 60.3 |
| motel | 51.7 | 59.5 | 56.6 |
| virus | 18.4 | 20.7 | 19.9 |
| infectious | 42.9 | 34.9 | 59.0 |
| outbreak | 7.8 | 8.1 | 7.7 |
| Rotavirus | 19.4 | 26.4 | 34.1 |
| Coronavirus | 6.0 | 6.1 | 10.3 |
| Influenza | 24.0 | 10.6 | 20.4 |
| food poisoning | 48.7 | 52.6 | 64.6 |
| incubation period | 19.4 | 21.2 | 14.2 |
| fever | 50.7 | 45.9 | 51.7 |
| poison | 41.8 | 57.6 | 60.0 |
| CDC | 32.1 | 30.4 | 31.7 |
| vaccine | 17.3 | 20.3 | 16.2 |
| Chipotle | 42.2 | 42.7 | 45.1 |
| ship | 49.9 | 51.9 | 49.8 |
| hand sanitizer | 25.6 | 27.5 | 26.6 |
| wash hand | 28.2 | 36 | 45.6 |
| flu symptom | 8.9 | 12.7 | 14.3 |
| streptococcus | 22.7 | 24.1 | 47.5 |
| antibiotics | 59.9 | 57.7 | 57.8 |
| candidiasis | 24.5 | 34.9 | 56.5 |
| otitis media | 19.0 | 38.9 | 55.8 |
| skin rash | 42.2 | 45.5 | 61.7 |
| Coxsackievirus | 13.1 | 13.4 | 25.5 |

Note: The number was normalized search volume data extracted from Google Trends website.
