# Supplementary material for: Predicting Norovirus in the United States Using Google Trends: Infodemiology Study
Source: J Med Internet Res. 2021 Sep 29;23(9):e24554. doi: 10.2196/24554 (PMC8515228; doi:10.2196/24554)
Supplement: Multimedia Appendix 2 [file jmir_v23i9e24554_app2.docx]

Multimedia Appendix 2. Cross-correlation analysis of norovirus and Internet search terms–New York.

| Search terms | Lags (month) | | | | | | |
| --- | --- | --- | --- | --- | --- | --- | --- |
|  | -3 | -2 | -1 | 0 | 1 | 2 | 3 |
| Internet search trends that were coincided with norovirus | | | | | | | |
| gastroenteritis | 0.077  *P*=.31 | 0.135  *P*=.07 | 0.236  *P*=.001 | 0.375  *P*<.001 | 0.319  *P*<.001 | 0.258  *P*<.001 | 0.192  *P*=.01 |
| watery diarrhea | 0.123  *P*=.10 | 0.182  *P*=.02 | 0.266  *P*<.001 | 0.339  *P*<.001 | 0.321  *P*<.001 | 0.250  *P*<.001 | 0.236  *P*=.002 |
| acute gastroenteritis | -0.063  *P*=.40 | 0.002  *P*=.98 | 0.113  *P*=.13 | 0.200  *P*=.007 | 0.075  *P*=.32 | 0.102  *P*=.18 | 0.100  *P*=.18 |
| stomach flu | 0.092  *P*=.22 | 0.281  *P*<.001 | 0.529  *P*<.001 | 0.636  *P*<.001 | 0.428  *P*<.001 | 0.271  *P*<.001 | 0.027  *P*=.72 |
| winter vomiting disease | 0.144  *P*=.05 | 0.177  *P*=.02 | 0.275  *P*<.001 | 0.365  *P*<.001 | 0.221  *P*=.003 | 0.020  *P*=.79 | -0.014  *P*=.85 |
| stomach bug | 0.113  *P*=.13 | 0.298  *P*<.001 | 0.537  *P*<.001 | 0.625  *P*<.001 | 0.470  *P*<.001 | 0.393  *P*<.001 | 0.199  *P*=.008 |
| food poisoning | 0.036  *P*=.63 | 0.191  *P*=.01 | 0.380  *P*<.001 | 0.416  *P*<.001 | 0.391  *P*<.001 | 0.302  *P*<.001 | 0.221  *P*=.003 |
| antibiotics | 0.276  *P*<.001 | 0.298  *P*<.001 | 0.313  *P*<.001 | 0.343  *P*<.001 | 0.311  *P*<.001 | 0.315  *P*<.001 | 0.247  *P*<.001 |
| fever | 0.110  *P*=.14 | 0.241  *P*=.001 | 0.382  *P*<.001 | 0.471  *P*<.001 | 0.456  *P*<.001 | 0.392  *P*<.001 | 0.308  *P*<.001 |
| Internet search trends earlier than norovirus | | | | | | | |
| contagious | 0.293  *P*<.001 | 0.347  *P*<.001 | 0.435  *P*<.001 | 0.433  *P*<.001 | 0.354  *P*<.001 | 0.280  *P*<.001 | 0.217  *P*=.004 |
| travel | -0.460  *P*<.001 | -0.410  *P*<.001 | -0.342  *P*<.001 | -0.270  *P*<.001 | -0.225  *P*=.002 | -0.218  *P*=.004 | -0.233  *P*=.002 |
| party | 0.374  *P*<.001 | 0.152  *P*=.04 | -0.002  *P*=.98 | -0.026  *P*=.73 | -0.024  *P*=.75 | 0.002  *P*=.98 | 0.118  *P*=.12 |
| cruise | -0.366  *P*<.001 | -0.310  *P*<.001 | -0.102  *P*=.17 | -0.028  *P*=.71 | 0.002  *P*=.98 | -0.025  *P*=.75 | 0.028  *P*=.72 |
| restaurant | -0.200  *P*=.008 | -0.121  *P*=.11 | -0.019  *P*=.80 | 0.091  *P*=.23 | 0.124  *P*=.10 | 0.136  *P*=.07 | 0.147  *P*=.05 |
| wedding | -0.327  *P*<.001 | -0.405  *P*<.001 | -0.370  *P*<.001 | -0.226  *P*=.002 | -0.086  *P*=.25 | -0.011  *P*=.88 | 0.163  *P*=.03 |
| hotel | -0.460  *P*<.001 | -0.486  *P*<.001 | -0.458  *P*<.001 | -0.361  *P*<.001 | -0.272  *P*<.001 | -0.210  *P*=.005 | -0.169  *P*=.02 |
| motel | -0.291  *P*<.001 | -0.313  *P*<.001 | -0.293  *P*<.001 | -0.171  *P*=.02 | -0.038  *P*=.62 | 0.140  *P*=.06 | 0.230  *P*=.002 |
| infectious | -0.137  *P*=.07 | -0.157  *P*=.04 | -0.166  *P*=.03 | -0.075  *P*=.32 | -0.092  *P*=.22 | -0.152  *P*=.04 | -0.112  *P*=.14 |
| incubation period | 0.035  *P*=.65 | 0.117  *P*=.12 | 0.207  *P*=.005 | 0.193  *P*=.009 | 0.136  *P*=.07 | 0.071  *P*=.35 | -0.011  *P*=.88 |
| vomiting | 0.138  *P*=.07 | 0.237  *P*=.001 | 0.416  *P*<.001 | 0.412  *P*<.001 | 0.366  *P*<.001 | 0.317  *P*<.001 | 0.165  *P*=.03 |
| norovirus infection | 0.088  *P*=.24 | 0.083  *P*=.27 | 0.166  *P*=.03 | 0.158  *P*=.03 | 0.100  *P*=.18 | -0.002  *P*=.98 | 0.006  *P*=.93 |
| poison | -0.236  *P*=.002 | -0.323  *P*<.001 | -0.328  *P*<.001 | -0.271  *P*<.001 | -0.173  *P*=.02 | 0.019  *P*=.80 | 0.194  *P*=.009 |
| CDC | -0.197  *P*=.009 | -0.209  *P*=.005 | -0.207  *P*=.005 | -0.171  *P*=.02 | -0.160  *P*=.03 | -0.190  *P*=.01 | -0.161  *P*=.03 |
| ship | 0.071  *P*=.35 | 0.128  *P*=.09 | 0.314  *P*<.001 | 0.207  *P*=.005 | 0.181  *P*=.02 | 0.148  *P*=.049 | 0.150  *P*=.046 |
| Internet search trends later than norovirus | | | | | | | |
| dehydration | 0.039  P=.61 | -0.027  P=.72 | 0.080  P=.28 | 0.076  P=.31 | 0.180  P=.02 | 0.163  P=.03 | 0.255  *P*<.001 |
| bar | 0.102  P=.18 | 0.125  P=.10 | 0.137  P=.07 | 0.165  P=.03 | 0.222  P=.003 | 0.281  *P*<.001 | 0.346  *P*<.001 |
| Noroviruses | -0.010  P=.89 | -0.003  *P*=.97 | 0.004  *P*=.95 | 0.027  *P*=.72 | 0.210  *P*=.005 | 0.140  *P*=.06 | -0.049  *P*=.51 |
| viral gastroenteritis | -0.033  *P*=.66 | -0.020  *P*=.79 | 0.029  *P*=.70 | 0.017  *P*=.82 | 0.030  *P*=.69 | 0.213  *P*=.004 | 0.207  *P*=.006 |
| barbecue | -0.195  *P*=.009 | -0.199  *P*=.008 | -0.157  *P*=.04 | -0.085  *P*=.26 | 0.018  *P*=.81 | 0.191  *P*=.01 | 0.383  *P*<.001 |
| oyster | 0.144  *P*=.06 | 0.019  *P*=.81 | -0.048  *P*=.53 | -0.000  *P*=.99 | 0.047  *P*=.53 | 0.143  *P*=.06 | 0.275  *P*<.001 |
| outbreak | 0.035  *P*=.64 | 0.089  *P*=.24 | 0.0701  *P*=.35 | 0.171  *P*=.02 | 0.316  *P*<.001 | 0.074  *P*=.32 | 0.002  *P*=.98 |
| coronavirus | 0.037  P=.63 | 0.076  P=.32 | 0.183  P=.01 | 0.321  *P*<.001 | 0.328  *P*<.001 | 0.339  *P*<.001 | 0.328  *P*<.001 |
| Chipotle | 0.297  *P*<.001 | 0.290  *P*<.001 | 0.293  *P*<.001 | 0.299  *P*<.001 | 0.312  *P*<.001 | 0.344  *P*<.001 | 0.350  *P*<.001 |
| hand sanitizer | 0.165  P=.03 | 0.164  P=.03 | 0.204  P=.006 | 0.226  P=.002 | 0.180  P=.02 | 0.231  P=.002 | 0.195  P=.009 |
| wash hand | 0.187  P=.01 | 0.199  P=.008 | 0.248  *P*<.001 | 0.280  *P*<.001 | 0.288  *P*<.001 | 0.317  *P*<.001 | 0.312  *P*<.001 |
| coxsackie virus | 0.066  P=.38 | 0.026  P=.73 | -0.028  P=.71 | -0.030  P=.69 | -0.002  P=.98 | 0.054  P=.48 | 0.263  *P*<.001 |

Note: R values represented cross correlation coefficient. P values represented statistical significance between two variables. Gray labeled values showed the maximum of cross correlation coefficient.
