# Supplementary material for: Predicting Norovirus in the United States Using Google Trends: Infodemiology Study
Source: J Med Internet Res. 2021 Sep 29;23(9):e24554. doi: 10.2196/24554 (PMC8515228; doi:10.2196/24554)
Supplement: Multimedia Appendix 3 [file jmir_v23i9e24554_app3.docx]

Multimedia Appendix 3. Cross-correlation analysis of actual norovirus cases and Internet search terms–New York.

| Search terms | Lags (month) | | | | | | |
| --- | --- | --- | --- | --- | --- | --- | --- |
|  | -3 | -2 | -1 | 0 | 1 | 2 | 3 |
| Internet search trends that were coincided with actual norovirus cases | | | | | | | |
| norovirus | -0.038  *P*=.62 | 0.120  *P*=.11 | 0.284  *P*<.001 | 0.362  *P*<.001 | 0.333  *P*<.001 | 0.188  *P*=.01 | -0.002  *P*=.98 |
| gastroenteritis | -0.025  *P*=.74 | 0.023  *P*=.76 | 0.144  *P*=.05 | 0.268  *P*<.001 | 0.261  *P*<.001 | 0.252  *P*<.001 | 0.191  *P*=.009 |
| norovirus infection | 0.060  *P*=.43 | 0.154  *P*=.04 | 0.248  *P*<.001 | 0.298  *P*<.001 | 0.071  *P*=.35 | 0.024  *P*=.75 | -0.073  *P*=.33 |
| contagious | 0.197  *P*=.007 | 0.226  *P*=.002 | 0.245  *P*<.001 | 0.255  *P*<.001 | 0.236  *P*=.001 | 0.164  *P*=.02 | 0.110  *P*=.13 |
| acute gastroenteritis | -0.014  *P*=.86 | 0.034  *P*=.65 | 0.040  *P*=.59 | 0.170  *P*=.02 | 0.111  *P*=.14 | 0.119  *P*=.11 | 0.159  *P*=.03 |
| stomach flu | -0.043  *P*=.57 | 0.194  *P*=.009 | 0.336  *P*<.001 | 0.416  *P*<.001 | 0.358  *P*<.001 | 0.188  *P*=.01 | 0.021  *P*=.78 |
| stomach bug | -0.071  *P*=.34 | 0.039  *P*=.60 | 0.203  *P*=.006 | 0.300  *P*<.001 | 0.283  *P*<.001 | 0.150  *P*=.04 | 0.045  *P*=.54 |
| food poisoning | -0.138  *P*=.07 | 0.032  *P*=.67 | 0.195  *P*=.009 | 0.260  *P*<.001 | 0.233  *P*=.001 | 0.121  *P*=.10 | 0.060  *P*=.41 |
| antibiotics | -0.007  *P*=.92 | 0.067  *P*=.37 | 0.172  *P*=.02 | 0.196  *P*=.008 | 0.187  *P*=.01 | 0.128  *P*=.09 | 0.084  *P*=.26 |
| Internet search trends earlier than actual norovirus cases | | | | | | | |
| travel | -0.370  *P*<.001 | -0.355  *P*<.001 | -0.292  *P*<.001 | -0.234  *P*=.002 | -0.211  *P*=.004 | -0.199  *P*=.006 | -0.214  *P*=.003 |
| party | 0.380  *P*<.001 | 0.160  *P*=.03 | -0.030  *P*=.69 | -0.096  *P*=.20 | -0.092  *P*=.22 | -0.039  *P*=.60 | 0.030  *P*=.69 |
| barbecue | -0.240  *P*=.001 | -0.253  *P*<.001 | -0.247  *P*<.001 | -0.206  *P*=.005 | -0.116  *P*=.12 | 0.011  *P*=.89 | 0.219  *P*=.003 |
| cruise | -0.316  *P*<.001 | -0.258  *P*<.001 | -0.125  *P*=.10 | 0.000  *P*=.99 | -0.018  *P*=.81 | -0.004  *P*=.95 | 0.020  *P*=.79 |
| oyster | 0.025  *P*=.73 | -0.094  *P*=.21 | -0.198  *P*=.008 | -0.192  *P*=.01 | -0.155  *P*=.04 | -0.055  *P*=.47 | 0.057  *P*=.44 |
| restaurant | -0.331  *P*<.001 | -0.245  *P*<.001 | -0.140  *P*=.06 | -0.014  *P*=.85 | 0.000  *P*=.99 | -0.028  *P*=.71 | -0.015  *P*=.84 |
| wedding | -0.267  *P*<.001 | -0.352  *P*<.001 | -0.342  *P*<.001 | -0.205  *P*=.006 | -0.088  *P*=.24 | 0.025  *P*=.74 | 0.199  *P*=.008 |
| hotel | -0.404  *P*<.001 | -0.445  *P*<.001 | -0.415  *P*<.001 | -0.337  *P*<.001 | -0.265  *P*<.001 | -0.212  *P*=.004 | -0.140  *P*=.05 |
| motel | -0.366  *P*<.001 | -0.441  *P*<.001 | -0.414  *P*<.001 | -0.318  *P*<.001 | -0.228  *P*=.002 | -0.084  *P*=.26 | 0.045  *P*=.55 |
| incubation period | 0.212  *P*=.005 | 0.251  *P*<.001 | 0.197  *P*=.008 | 0.135  *P*=.07 | 0.075  *P*=.32 | 0.123  *P*=.09 | 0.110  *P*=.13 |
| poison | -0.169  *P*=.02 | -0.299  *P*<.001 | -0.303  *P*<.001 | -0.289  *P*<.001 | -0.161  *P*=.03 | 0.007  *P*=.92 | 0.173  *P*=.02 |
| vaccine | 0.341  *P*<.001 | 0.216  *P*=.004 | 0.058  *P*=.44 | -0.053  *P*=.48 | -0.080  *P*=.29 | -0.109  *P*=.14 | -0.084  *P*=.26 |
| hand sanitizer | 0.161  *P*=.03 | 0.128  *P*=.09 | 0.076  *P*=.31 | 0.046  *P*=.54 | 0.083  *P*=.26 | 0.117  *P*=.12 | 0.131  *P*=.08 |
| skin rash | -0.157  *P*=.04 | -0.117  *P*=.12 | -0.126  *P*=.09 | -0.077  *P*=.30 | -0.008  *P*=.92 | -0.022  *P*=.77 | 0.022  *P*=.76 |
| Internet search trends later than actual norovirus cases | | | | | | | |
| vomiting | -0.005  *P*=.95 | 0.082  *P*=.27 | 0.151  *P*=.04 | 0.197  *P*=.008 | 0.226  *P*=.002 | 0.155  *P*=.04 | -0.024  *P*=.75 |
| dehydration | -0.193  *P*=.01 | -0.183  *P*=.01 | -0.130  *P*=.08 | -0.038  *P*=.61 | 0.016  *P*=.83 | 0.115  *P*=.11 | 0.221  *P*=.002 |
| winter vomiting disease | 0.011  *P*=.83 | 0.071  *P*=.31 | 0.140  *P*=.05 | 0.156  *P*=.03 | 0.163  *P*=.03 | 0.076  *P*=.29 | -0.018  *P*=.85 |
| outbreak | -0.050  *P*=.51 | -0.010  *P*=.89 | 0.052  *P*=.49 | 0.091  *P*=.22 | 0.166  *P*=.03 | 0.134  *P*=.07 | 0.049  *P*=.51 |
| rotavirus | 0.000  *P*=.99 | -0.014  *P*=.85 | 0.040  *P*=.59 | 0.149  *P*=.046 | 0.179  *P*=.02 | 0.154  *P*=.04 | 0.076  *P*=.30 |
| coronavirus | -0.064  *P*=.40 | -0.060  *P*=.43 | -0.034  *P*=.65 | 0.012  *P*=.87 | 0.129  *P*=.09 | 0.166  *P*=.03 | 0.128  *P*=.09 |
| fever | -0.036  *P*=.63 | 0.028  *P*=.71 | 0.100  *P*=.18 | 0.191  *P*=.01 | 0.290  *P*<.001 | 0.231  *P*=.002 | 0.162  *P*=.03 |

Note: R values represented cross correlation coefficient. P values represented statistical significance between two variables. Gray labeled values showed the maximum of cross correlation coefficient.
