# Supplementary material for: Predicting Norovirus in the United States Using Google Trends: Infodemiology Study
Source: J Med Internet Res. 2021 Sep 29;23(9):e24554. doi: 10.2196/24554 (PMC8515228; doi:10.2196/24554)
Supplement: Multimedia Appendix 4 [file jmir_v23i9e24554_app4.docx]

Multimedia Appendix 4. Cross-correlation analysis of actual norovirus cases and Internet search terms–California.

| Search terms | Lags (month) | | | | | | |
| --- | --- | --- | --- | --- | --- | --- | --- |
|  | -3 | -2 | -1 | 0 | 1 | 2 | 3 |
| Internet search trends that were coincided with actual norovirus cases | | | | | | | |
| waterydiarrhea | -0.208  *P*=.005 | -0.201  *P*=.007 | -0.238  *P*=.001 | -0.253  *P*<.001 | -0.248  *P*<.001 | -0.225  *P*=.003 | -0.202  *P*=.007 |
| Internet search trends earlier than actual norovirus cases | | | | | | | |
| barbecue | -0.251  *P*<.001 | -0.300  *P*<.001 | -0.265  *P*<.001 | -0.206  *P*=.006 | -0.160  *P*=.03 | -0.149  *P*=.047 | -0.095  *P*=.21 |
| oyster | -0.199  *P*=.008 | -0.203  *P*=.007 | -0.175  *P*=.02 | -0.140  *P*=.06 | -0.142  *P*=.06 | -0.179  *P*=.02 | -0.150  *P*=.045 |
| bar | -0.165  *P*=.03 | -0.176  *P*=.02 | -0.151  *P*=.04 | -0.160  *P*=.03 | -0.172  *P*=.02 | -0.138  *P*=.07 | -0.134  *P*=.07 |
| infectious | 0.129  *P*=.09 | 0.256  *P*<.001 | 0.199  *P*=.008 | 0.188  *P*=.01 | 0.180  *P*=.02 | 0.247  *P*<.001 | 0.249  *P*<.001 |
| food poisoning | -0.203  *P*=.007 | -0.190  *P*=.01 | -0.058  *P*=.44 | 0.003  *P*=.96 | -0.036  *P*=.63 | -0.124  *P*=.10 | -0.200  *P*=.008 |
| CDC | 0.126  *P*=.09 | 0.176  *P*=.02 | 0.204  *P*=.006 | 0.097  *P*=.20 | 0.089  *P*=.23 | 0.100  *P*=.19 | 0.187  *P*=.01 |
| Chipotle | -0.306  *P*<.001 | -0.322  *P*<.001 | -0.297  *P*<.001 | -0.297  *P*<.001 | -0.311  *P*<.001 | -0.309  *P*<.001 | -0.287  *P*<.001 |
| streptococcus | -0.050  *P*=.51 | 0.177  *P*=.02 | 0.147  *P*=.049 | 0.175  *P*=.02 | 0.124  *P*=.10 | 0.082  *P*=.27 | 0.034  *P*=.65 |
| skin rash | -0.138  *P*=.07 | -0.235  *P*=.002 | -0.084  *P*=.26 | -0.133  *P*=.08 | -0.063  *P*=.40 | -0.035  *P*=.64 | 0.018  *P*=.81 |
| gastroenteritis | -0.198  *P*=.008 | -0.184  *P*=.01 | -0.190  *P*=.01 | -0.108  *P*=.15 | -0.071  *P*=.35 | -0.164  *P*=.03 | -0.084  *P*=.27 |
| Internet search trends later than actual norovirus cases | | | | | | | |
| diarrhea | -0.268  *P*<.001 | -0.332  *P*<.001 | -0.331  *P*<.001 | -0.311  *P*<.001 | -0.306  *P*<.001 | -0.317  *P*<.001 | -0.332  *P*<.001 |
| vomiting | -0.166  *P*=.03 | -0.182  *P*=.01 | -0.189  *P*=.01 | -0.149  *P*=.046 | -0.215  *P*=.004 | -0.252  *P*<.001 | -0.299  *P*<.001 |
| contagious | -0.276  *P*<.001 | -0.283  *P*<.001 | -0.277  *P*<.001 | -0.305  *P*<.001 | -0.363  *P*<.001 | -0.368  *P*<.001 | -0.410  *P*<.001 |
| contaminated water | 0.136  *P*=.07 | 0.185  *P*=.01 | 0.017  *P*=.82 | -0.084  *P*=.26 | 0.024  *P*=.75 | 0.027  *P*=.72 | 0.198  *P*=.008 |
| norwalk virus | 0.112  *P*=.14 | 0.125  *P*=.10 | 0.321  *P*<.001 | 0.360  *P*<.001 | 0.410  *P*<.001 | 0.186  *P*=.01 | 0.214  *P*=.004 |
| stomach flu | -0.149  *P*=.049 | -0.079  *P*=.30 | -0.015  *P*=.84 | 0.058  *P*=.44 | -0.032  *P*=.67 | -0.171  *P*=.02 | -0.255  *P*<.001 |
| stomach bug | -0.183  *P*=.01 | -0.186  *P*=.01 | -0.180  *P*=.02 | -0.146  *P*=.05 | -0.190  *P*=.01 | -0.253  *P*<.001 | -0.309  *P*<.001 |
| travel | 0.346  *P*<.001 | 0.333  *P*<.001 | 0.325  *P*<.001 | 0.366  *P*<.001 | 0.414  *P*<.001 | 0.437  *P*<.001 | 0.444  *P*<.001 |
| party | -0.109  *P*=.15 | 0.012  *P*=.87 | -0.101  *P*=.18 | -0.216  *P*=.004 | -0.286  *P*<.001 | -0.282  *P*<.001 | -0.228  *P*=.002 |
| cruise | 0.118  *P*=.12 | 0.108  *P*=.15 | 0.042  *P*=.57 | 0.169  *P*=.02 | 0.291  *P*<.001 | 0.287  *P*<.001 | 0.277  *P*<.001 |
| restaurant | 0.157  *P*=.04 | 0.109  *P*=.15 | 0.167  *P*=.03 | 0.222  *P*=.003 | 0.237  *P*=.001 | 0.237  *P*=.001 | 0.175  *P*=.02 |
| wedding | 0.000  *P*=.99 | -0.043  *P*=.57 | -0.086  *P*=.25 | -0.023  *P*=.76 | 0.086  *P*=.25 | 0.197  *P*=.008 | 0.143  *P*=.06 |
| hotel | 0.338  *P*<.001 | 0.313  *P*<.001 | 0.277  *P*<.001 | 0.308  *P*<.001 | 0.389  *P*<.001 | 0.454  *P*<.001 | 0.469  *P*<.001 |
| virus | 0.155  *P*=.04 | 0.102  *P*=.18 | 0.042  *P*=.58 | 0.006  *P*=.94 | 0.082  *P*=.27 | 0.159  *P*=.03 | 0.143  *P*=.06 |
| incubation period | -0.094  *P*=.21 | -0.003  *P*=.97 | -0.028  *P*=.71 | -0.171  *P*=.02 | -0.185  *P*=.01 | -0.203  *P*=.006 | -0.196  *P*=.009 |
| fever | -0.129  *P*=.09 | -0.085  *P*=.26 | -0.133  *P*=.08 | -0.155  *P*=.04 | -0.171  *P*=.02 | -0.094  *P*=.21 | -0.146  *P*=.05 |
| poison | -0.008  *P*=.92 | 0.119  *P*=.11 | 0.070  *P*=.35 | 0.138  *P*=.06 | 0.070  *P*=.35 | 0.191  *P*=.01 | 0.159  *P*=.03 |
| vaccine | -0.105  *P*=.16 | -0.022  *P*=.77 | -0.096  *P*=.20 | -0.208  *P*=.005 | -0.304  *P*<.001 | -0.248  *P*<.001 | -0.228  *P*=.002 |
| ship | -0.180  *P*=.02 | -0.120  *P*=.11 | -0.141  *P*=.06 | -0.165  *P*=.03 | -0.189  *P*=.01 | -0.207  *P*=.005 | -0.165  *P*=.03 |
| hand sanitizer | -0.241  *P*=.001 | -0.149  *P*=.047 | -0.240  *P*=.001 | -0.289  *P*<.001 | -0.371  *P*<.001 | -0.404  *P*<.001 | -0.328  *P*<.001 |
| wash hand | -0.251  *P*<.001 | -0.277  *P*<.001 | -0.260  *P*<.001 | -0.280  *P*<.001 | -0.309  *P*<.001 | -0.245  *P*=.001 | -0.229  *P*=.002 |
| antibiotics | -0.253  *P*<.001 | -0.242  *P*=.001 | -0.235  *P*=.002 | -0.266  *P*<.001 | -0.306  *P*<.001 | -0.261  *P*<.001 | -0.256  *P*<.001 |
| otitis media | -0.062  *P*=.41 | -0.041  *P*=.59 | 0.164  *P*=.03 | 0.030  *P*=.68 | 0.173  *P*=.02 | 0.092  *P*=.22 | 0.182  *P*=.02 |
| coxsackie virus | -0.184  *P*=.01 | -0.114  *P*=.13 | -0.203  *P*=.007 | -0.121  *P*=.10 | -0.206  *P*=.006 | -0.100  *P*=.19 | -0.133  *P*=.08 |

Note: R values represented cross correlation coefficient. P values represented statistical significance between two variables. Gray labeled values showed the maximum of cross correlation coefficient.
