# Supplementary material for: Predicting Norovirus in the United States Using Google Trends: Infodemiology Study
Source: J Med Internet Res. 2021 Sep 29;23(9):e24554. doi: 10.2196/24554 (PMC8515228; doi:10.2196/24554)
Supplement: Multimedia Appendix 5 [file jmir_v23i9e24554_app5.docx]

Multimedia Appendix 5. Cross-correlation analysis of actual norovirus cases and Internet search terms–US.

| Search terms | Lags (month) | | | | | | |
| --- | --- | --- | --- | --- | --- | --- | --- |
|  | -3 | -2 | -1 | 0 | 1 | 2 | 3 |
| Internet search trends that were coincided with actual norovirus cases | | | | | | | |
| vomiting | 0.309  *P*<.001 | 0.425  *P*<.001 | 0.537  *P*<.001 | 0.609  *P*<.001 | 0.572  *P*<.001 | 0.489  *P*<.001 | 0.402  *P*<.001 |
| contaminated | 0.214  *P*=.004 | 0.128  *P*=.09 | 0.100  *P*=.18 | 0.158  *P*=.03 | 0.104  *P*=.17 | 0.107  *P*=.15 | 0.133  *P*=.08 |
| norovirus infection | 0.157  *P*=.04 | 0.384  *P*<.001 | 0.501  *P*<.001 | 0.595  *P*<.001 | 0.515  *P*<.001 | 0.263  *P*<.001 | 0.084  *P*=.27 |
| contagious | 0.476  *P*<.001 | 0.548  *P*<.001 | 0.580  *P*<.001 | 0.587  *P*<.001 | 0.548  *P*<.001 | 0.439  *P*<.001 | 0.337  *P*<.001 |
| acute gastroenteritis | -0.071  *P*=.35 | -0.000  *P*=.99 | 0.098  *P*=.19 | 0.204  *P*=.006 | 0.200  *P*=.007 | 0.196  *P*=.009 | 0.167  *P*=.03 |
| stomach flu | 0.182  *P*=.02 | 0.465  *P*<.001 | 0.687  *P*<.001 | 0.786  *P*<.001 | 0.658  *P*<.001 | 0.372  *P*<.001 | 0.104  *P*=.17 |
| viral gastroenteritis | -0.022  *P*=.77 | 0.153  *P*=.04 | 0.256  *P*<.001 | 0.345  *P*<.001 | 0.300  *P*<.001 | 0.164  *P*=.03 | 0.032  *P*=.67 |
| stomach bug | 0.242  *P*=.001 | 0.407  *P*<.001 | 0.581  *P*<.001 | 0.690  *P*<.001 | 0.628  *P*<.001 | 0.456  *P*<.001 | 0.300  *P*<.001 |
| food poisoning | 0.107  *P*=.16 | 0.298  *P*<.001 | 0.510  *P*<.001 | 0.633  *P*<.001 | 0.619  *P*<.001 | 0.531  *P*<.001 | 0.454  *P*<.001 |
| incubation period | 0.202  *P*=.007 | 0.232  *P*=.002 | 0.236  *P*=.001 | 0.252  *P*<.001 | 0.220  *P*=.003 | 0.099  *P*=.19 | -0.007  *P*=.93 |
| CDC | -0.091  *P*=.23 | -0.165  *P*=.03 | -0.243  *P*=.001 | -0.251  *P*<.001 | -0.205  *P*=.006 | -0.187  *P*=.01 | -0.152  *P*=.04 |
| ship | 0.146  *P*=.05 | 0.207  *P*=.005 | 0.330  *P*<.001 | 0.375  *P*<.001 | 0.337  *P*<.001 | 0.287  *P*<.001 | 0.312  *P*<.001 |
| flu symptom | 0.144  *P*=.06 | 0.228  *P*=.002 | 0.259  *P*<.001 | 0.276  *P*<.001 | 0.252  *P*<.001 | 0.112  *P*=.14 | -0.022  *P*=.77 |
| antibiotics | 0.368  *P*<.001 | 0.434  *P*<.001 | 0.481  *P*<.001 | 0.531  *P*<.001 | 0.529  *P*<.001 | 0.476  *P*<.001 | 0.409  *P*<.001 |
| candidiasis | -0.073  *P*=.33 | -0.081  *P*=.28 | -0.151  *P*=.04 | -0.153  *P*=.04 | -0.133  *P*=.08 | -0.122  *P*=.10 | -0.113  *P*=.13 |
| otitis media | 0.092  *P*=.22 | 0.206  *P*=.006 | 0.281  *P*<.001 | 0.332  *P*<.001 | 0.320  *P*<.001 | 0.197  *P*=.008 | 0.008  *P*=.92 |
| Internet search trends earlier than actual norovirus cases | | | | | | | |
| travel | -0.557  *P*<.001 | -0.527  *P*<.001 | -0.464  *P*<.001 | -0.391  *P*<.001 | -0.340  *P*<.001 | -0.313  *P*<.001 | -0.304  *P*<.001 |
| party | 0.509  *P*<.001 | 0.284  *P*<.001 | 0.048  *P*=.53 | -0.046  *P*=.54 | -0.057  *P*=.44 | -0.035  *P*=.65 | 0.017  *P*=.83 |
| cruise | -0.482  *P*<.001 | -0.390  *P*<.001 | -0.189  *P*=.01 | 0.017  *P*=.82 | 0.075  *P*=.32 | 0.050  *P*=.51 | 0.042  *P*=.58 |
| restaurant | -0.199  *P*=.008 | -0.165  *P*=.03 | -0.045  *P*=.55 | 0.063  *P*=.39 | 0.099  *P*=.19 | 0.122  *P*=.11 | 0.177  *P*=.02 |
| wedding | -0.443  *P*<.001 | -0.515  *P*<.001 | -0.470  *P*<.001 | -0.310  *P*<.001 | -0.150  *P*=.04 | -0.011  *P*=.88 | 0.132  *P*=.08 |
| hotel | -0.586  *P*<.001 | -0.613  *P*<.001 | -0.578  *P*<.001 | -0.493  *P*<.001 | -0.412  *P*<.001 | -0.341  *P*<.001 | -0.280  *P*<.001 |
| motel | -0.389  *P*<.001 | -0.481  *P*<.001 | -0.414  *P*<.001 | -0.242  *P*=.001 | -0.040  *P*=.60 | 0.155  *P*=.04 | 0.310  *P*<.001 |
| infectious | -0.290  *P*<.001 | -0.297  *P*<.001 | -0.295  *P*<.001 | -0.232  *P*=.002 | -0.133  *P*=.08 | -0.124  *P*=.10 | -0.163  *P*=.03 |
| vaccine | 0.320  *P*<.001 | 0.193  *P*=.01 | 0.053  *P*=.48 | -0.012  *P*=.88 | -0.046  *P*=.54 | -0.063  *P*=.40 | -0.050  *P*=.51 |
| poison | -0.335  *P*<.001 | -0.519  *P*<.001 | -0.592  *P*<.001 | -0.511  *P*<.001 | -0.294  *P*<.001 | 0.013  P=.86 | 0.337  *P*<.001 |
| hand sanitizer | 0.339  *P*<.001 | 0.296  *P*<.001 | 0.257  *P*<.001 | 0.246  *P*<.001 | 0.248  *P*<.001 | 0.250  *P*<.001 | 0.250  *P*<.001 |
| streptococcus | -0.198  *P*=.008 | -0.140  *P*=.06 | -0.147  *P*=.049 | -0.112  *P*=.13 | -0.038  *P*=.62 | -0.037  *P*=.63 | -0.124  *P*=.10 |
| Internet search trends later than actual norovirus cases | | | | | | | |
| gastroenteritis | 0.190  *P*=.01 | 0.345  *P*<.001 | 0.502  *P*<.001 | 0.630  *P*<.001 | 0.641  *P*<.001 | 0.547  *P*<.001 | 0.409  *P*<.001 |
| diarrhea | 0.324  *P*<.001 | 0.327  *P*<.001 | 0.357  *P*<.001 | 0.405  *P*<.001 | 0.424  *P*<.001 | 0.435  *P*<.001 | 0.454  *P*<.001 |
| dehydration | 0.080  *P*=.29 | 0.031  *P*=.68 | 0.034  *P*=.66 | 0.114  *P*=.13 | 0.207  *P*=.005 | 0.306  *P*<.001 | 0.440  *P*<.001 |
| watery diarrhea | 0.260  *P*<.001 | 0.313  *P*<.001 | 0.399  *P*<.001 | 0.458  *P*<.001 | 0.475  *P*<.001 | 0.471  *P*<.001 | 0.461  *P*<.001 |
| contaminated water | 0.075  *P*=.32 | 0.027  *P*=.72 | 0.022  *P*=.77 | 0.105  *P*=.16 | 0.147  *P*=.05 | 0.210  *P*=.005 | 0.259  *P*<.001 |
| barbecue | 0.005  *P*=.95 | -0.051  *P*=.50 | -0.018  *P*=.81 | 0.067  *P*=.37 | 0.199  *P*=.008 | 0.378  *P*<.001 | 0.563  *P*<.001 |
| oyster | 0.236  *P*=.002 | 0.191  *P*=.01 | 0.175  *P*=.02 | 0.184  *P*=.01 | 0.220  *P*=.003 | 0.303  *P*<.001 | 0.397  *P*<.001 |
| bar | 0.184  *P*=.01 | 0.186  *P*=.01 | 0.210  *P*=.005 | 0.251  *P*<.001 | 0.270  *P*<.001 | 0.308  *P*<.001 | 0.364  *P*<.001 |
| virus | -0.035  *P*=.64 | -0.133  *P*=.08 | -0.174  *P*=.02 | -0.182  *P*=.01 | -0.170  *P*=.02 | -0.200  *P*=.007 | -0.185  *P*=.01 |
| rotavirus | -0.220  *P*=.003 | -0.067  *P*=.37 | 0.103  *P*=.17 | 0.223  *P*=.003 | 0.267  *P*<.001 | 0.172  *P*=.02 | 0.030  *P*=.69 |
| coronavirus | -0.070  *P*=.35 | 0.019  *P*=.80 | 0.169  *P*=.02 | 0.275  *P*<.001 | 0.359  *P*<.001 | 0.363  *P*<.001 | 0.292  *P*<.001 |
| influenza | -0.030  *P*=.69 | 0.080  *P*=.29 | 0.126  *P*=.09 | 0.219  *P*=.003 | 0.264  *P*<.001 | 0.117  *P*=.12 | -0.061  *P*=.42 |
| fever | 0.193  *P*=.01 | 0.305  *P*<.001 | 0.411  *P*<.001 | 0.555  *P*<.001 | 0.623  *P*<.001 | 0.514  *P*<.001 | 0.351  *P*<.001 |
| Chipotle | 0.353  *P*<.001 | 0.349  *P*<.001 | 0.350  *P*<.001 | 0.369  *P*<.001 | 0.386  *P*<.001 | 0.414  *P*<.001 | 0.446  *P*<.001 |
| wash hand | 0.260  *P*<.001 | 0.235  *P*=.002 | 0.238  *P*=.001 | 0.289  *P*<.001 | 0.312  *P*<.001 | 0.352  *P*<.001 | 0.419  *P*<.001 |
| skin rash | -0.075  *P*=.32 | -0.141  *P*=.06 | -0.087  *P*=.25 | 0.027  *P*=.72 | 0.140  *P*=.06 | 0.286  *P*<.001 | 0.437  *P*<.001 |
| coxsackie virus | -0.131  *P*=.08 | -0.249  *P*<.001 | -0.386  *P*<.001 | -0.446  *P*<.001 | -0.448  *P*<.001 | -0.383  *P*<.001 | -0.266  *P*<.001 |

Note: R values represented cross correlation coefficient. P values represented statistical significance between two variables. Gray labeled values showed the maximum of cross correlation coefficient.
